# Exercise cardiovascular magnetic resonance myocardial dynamic index: A non-invasive imaging marker associated with cardiac dyspnea

**DOI:** 10.1016/j.jocmr.2025.101981

**Published:** 2025-10-27

**Authors:** Fahime Ghanbari, Jennifer Rodriguez, Manuel A. Morales, Long H. Ngo, Connie W. Tsao, Jeremy M. Robbins, Deepa M. Gopal, David M. Systrom, Aaron B. Waxman, Warren J. Manning, Reza Nezafat

**Affiliations:** a Departments of Medicine (Cardiovascular Division) Beth Israel Deaconess Medical Center and Harvard Medical School, Boston, Massachusetts, USA; b Departments of Radiology, Beth Israel Deaconess Medical Center and Harvard Medical School, Boston, Massachusetts, USA; c Division of Cardiovascular Medicine, Boston University Chobanian & Avedisian School of Medicine, Boston, Massachusetts, USA; d Department of Medicine (Division of Pulmonary and Critical Care), Brigham and Women's Hospital and Harvard Medical School, Boston, Massachusetts, USA

**Keywords:** Exercise-CMR, Work-volume loop model, Myocardial dynamic index, Non-invasive imaging marker, Cardiac dyspnea, VO_2_ max index

## Abstract

**Background:**

Identifying the cause of dyspnea (i.e., cardiac vs. non-cardiac) can be challenging in the absence of significant resting cardiac abnormalities. Exercise cardiovascular magnetic resonance (Ex-CMR) enables quantification of cardiac volumetric indices under physiological stress. Using Ex-CMR, we sought to develop a non-invasive imaging marker, referred to as the myocardial dynamic index (MDI), and to demonstrate its potential for evaluating cardiac dyspnea.

**Methods:**

MDI is a metric derived from Ex-CMR work-volume loop model that integrates rest and stress left ventricular (LV) end-diastolic and end-systolic volumes with workload measured during supine exercise, while accounting for body size and LV mass. To evaluate MDI as a marker of cardiac dyspnea, we retrospectively analyzed data from a prospective multicenter study measuring MDI in patients with cardiac or non-cardiac dyspnea. All had invasive exercise testing before Ex-CMR. Cardiac dyspnea was defined by established invasive and non-invasive criteria, including HFpEF (early to advanced) and HFmrEF. Non-cardiac dyspnea patients had normal invasive hemodynamics and cardiac function. Univariable and multivariable logistic regression identified clinical and imaging predictors of cardiac dyspnea. A base model incorporating clinical and rest CMR variables was compared to a model that included the base model plus MDI. Diagnostic performance was assessed using receiver operating characteristic analysis and compared using the DeLong test. MDI scan/re-scan reproducibility over one year, inter- and intra-observer reproducibility, and correlation with VO₂ max were evaluated.

**Results:**

Among 93 patients (66 with cardiac dyspnea, 27 with non-cardiac dyspnea), MDI was lower in patients with cardiac dyspnea (25.9 ± 9.5 vs. 45.1 ± 10.7 mL·W/g/m², p<0.0001). The base model included age, body mass index, NYHA class, and left atrial strain. In multivariable analysis, MDI emerged as the only independent predictor of cardiac dyspnea when added to the base model. Inclusion of MDI improved the AUC from 0.86 to 0.93 (p = 0.012), while MDI alone yielded an AUC of 0.91. A strong correlation was observed between MDI and the VO₂ max index (r = 0.84, p<0.0001). Reproducibility was excellent.

**Conclusion:**

Ex-CMR MDI is independently associated with cardiac dyspnea and strongly correlates with the VO₂ max index. It aids in differentiating cardiac from non-cardiac dyspnea and provides incremental diagnostic value beyond conventional clinical and resting imaging parameters.

## Graphical abstract

**Figure:**
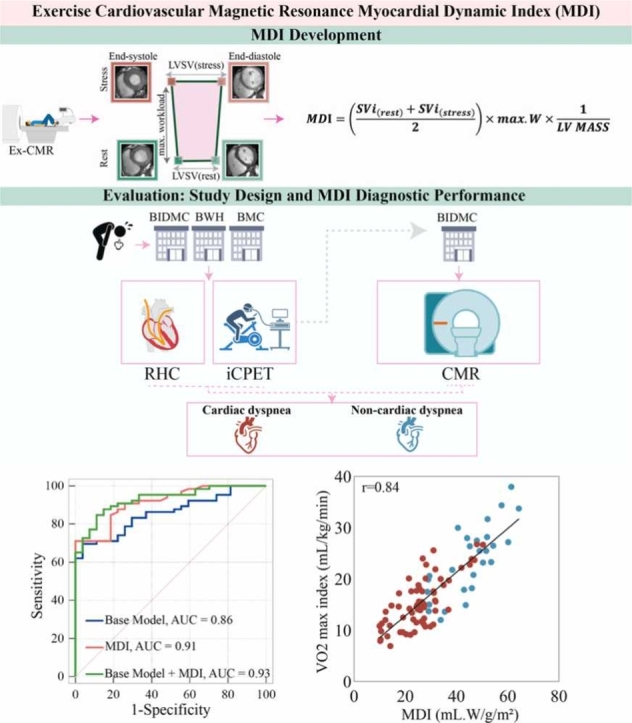
The MDI is derived from the Ex-CMR work–volume loop area equation, adjusted for myocardial mass and body surface area. MDI was evaluated in 93 patients with cardiac or non-cardiac dyspnea and demonstrated added value beyond conventional clinical and resting CMR variables, along with a strong correlation with the VO₂ max index. *Ex-CMR* exercise cardiovascular magnetic resonance, *iCEPT* invasive cardiopulmonary exercise testing, *max.W* maximum workload achieved within 10 min of supine Ex-CMR, *RHC* right heart catheterization, *SVi* stroke volume index

## 1 Introduction

Dyspnea is a prevalent and debilitating symptom, but identifying its underlying cause can be challenging, requiring a multidisciplinary approach that often leads to prolonged referral delays [1], [2]. Cardiac dyspnea, characterized by exertional dyspnea, fatigue, and exercise intolerance, is particularly challenging to identify when conventional tests reveal no overt heart failure, ischemia, or obstructive pathology, and biomarkers remain within normal limits [3], [4], [5]. Furthermore, non-cardiac conditions frequently mimic cardiac symptoms, further complicating diagnosis [6]. Evidence of elevated left ventricular (LV) filling pressures at rest or during stress is a key pathophysiological hallmark of cardiac dyspnea, particularly in patients spanning the spectrum of heart failure with preserved ejection fraction (HFpEF) (overt or exercise-induced) and HF with mildly reduced EF (HFmrEF) [4]. However, non-invasive resting modalities may not reliably detect increased filling pressures, necessitating invasive hemodynamic assessment during exercise, which is challenging to use routinely. Furthermore, current guidelines classify patients with preserved LVEF but with structural or functional abnormalities, such as reduced LV longitudinal strain or mild concentric remodeling on resting imaging, as having stage B HFpEF [4], [7]. These patients may present with exertional dyspnea despite normal invasive hemodynamics, making diagnosis challenging. Stage B HFpEF is often overlooked due to mild symptoms and limited diagnostic sensitivity, despite representing a key opportunity for early detection and intervention [8]. Currently, a non-invasive imaging marker that can differentiate cardiac from non-cardiac causes of dyspnea before escalating the work-up to invasive testing and that reflects oxygen consumption under physiological stress is lacking and represents an important knowledge gap.

Exercise increases cardiomyocyte oxygen demand and alters preload and afterload, requiring diagnostic modalities that capture cardiac adaptations [9]. While invasive pressure-volume (PV) analysis during exercise provides critical insights, its clinical applicability is limited [10], [11], [12], [13], [14]. Cardiovascular magnetic resonance (CMR) is the gold standard for non-invasive cardiac volume evaluation and offers valuable structural assessment [4], [15]. Exercise-CMR (Ex-CMR) is a promising technique for capturing dynamic volumetric changes during exercise and revealing abnormalities not evident at rest [16]. Most recent advances in CMR have enabled highly accelerated, free-breathing imaging of the entire heart in less than a minute at high spatiotemporal resolutions, with excellent reproducibility and good quality [17], [18]. However, the full potential of Ex-CMR-derived data to develop a non-invasive imaging marker for evaluating cardiac dyspnea remains underexplored.

Cardio-hemodynamic principles have long guided our understanding of cardiac pathologies, with invasive PV loop area reflecting myocardial external work and oxygen consumption [19], [20], [21], [22]. This study proposes the Ex-CMR myocardial dynamic index (MDI), a novel imaging marker that integrates rest and stress LV volumes with workload (measured in watts during supine exercise) through a new work-volume loop framework. Additionally, we sought to evaluate its diagnostic performance in differentiating patients with cardiac dyspnea from non-cardiac dyspnea, as well as its correlation with maximum oxygen uptake (VO_2_ max index).

## 2 Methods

### 2.1 Myocardial dynamic index development

**Fig. 1:**
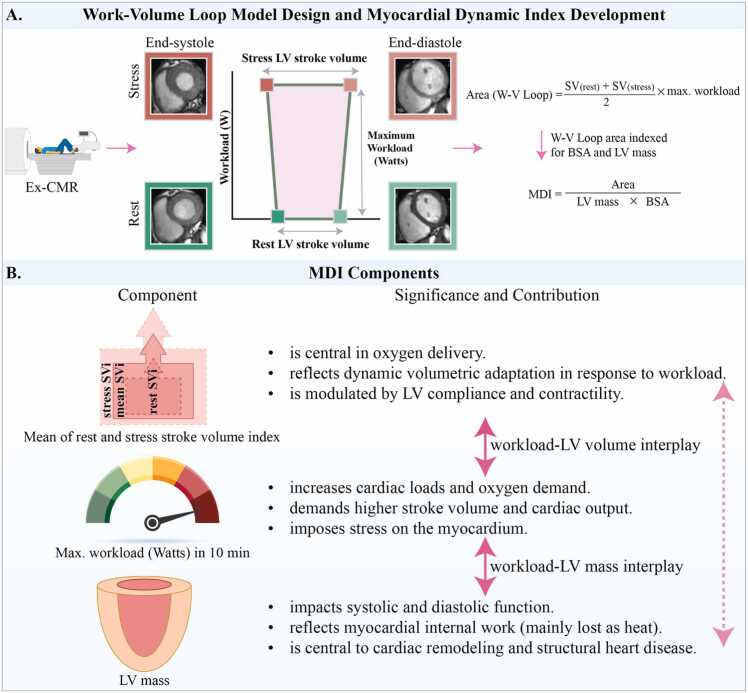
Work-volume loop model design and myocardial dynamic index development. Panel A shows the work-volume loop model design and MDI development. Panel B illustrates the MDI components, their significance, and the interaction among its three pillars. *BSA* body surface area, *Ex-CMR* exercise cardiovascular magnetic resonance, *LV* left ventricle, *MDI* myocardial dynamic index, *SVi* stroke volume index

The MDI is derived from a non-invasive work-volume (W-V) loop derived from Ex-CMR (Fig. 1**A**). The W-V loop, which forms a trapezoidal shape, is based on five key parameters: LV end-diastolic volumes (LVEDV) and end-systolic volumes (LVESV) at rest and stress, along with the maximum achieved workload (W), measured in watts during the supine bike exercise session. This workload is a key driver of the volumetric changes between rest and stress, reflecting the physiological load placed on the heart during exercise. Although it does not necessarily represent the patient's absolute maximum exercise capacity, it is the highest documented workload achieved within 10 min of supine exercise. In the X-Y plot, LVEDV and LVESV are represented on the X-axis, while W is plotted on the Y-axis. The upper and lower sides of the trapezoid corresponded to stroke volumes (SV) at rest and stress, respectively, and its height reflected the W. The area of the trapezoid is calculated by substituting these data into the area equation. SVs are indexed to body surface area (SVi), and the equation is adjusted for myocardial mass (LV mass), resulting in the following formula:

$$\mathrm{MDI}=\left(\frac{{\mathrm{SVi}}_{\left(\mathrm{rest}\right)}+{\mathrm{SVi}}_{\left(\mathrm{stress}\right)}}{2}\right)\times W\times 1/{\mathrm{LV}}_{\mathrm{mass}}$$

Fig. 1**B** illustrates the components of MDI and their pathophysiological contributions. The beating heart operates dynamically through temporally and spatially coordinated contraction and relaxation of cardiomyocytes to meet oxygen demands and performs both external (stroke work) and internal work. External work drives volumetric dynamics and ejects blood, while internal work modifies myocardial tissue mechanics, much of which dissipates as heat [20], [21], [23]. Traditional assessments of myocardial work or its surrogates require invasive pressure measurements or estimates of wall stress [19], [20]. Instead of estimating myocardial work, we propose MDI as a non-invasive metric that quantifies myocardial dynamics relative to workload. This metric integrates the interplay of three key components into a single measure (Fig. 1).

### 2.2 Evaluation

We measured MDI in patients with cardiac or non-cardiac dyspnea, assessed its effectiveness in differentiating these groups, examined its additive value beyond conventional clinical and resting CMR variables, and evaluated its correlation with the VO₂ max index from invasive cardiopulmonary exercise testing (iCPET).

### 2.3 Study design and patient population

This HIPAA-compliant study was approved by our Institutional Review Board. We performed a retrospective analysis of a prospective, multicenter study that enrolled dyspneic patients with cardiac or non-cardiac dyspnea from Beth Israel Deaconess Medical Center (BIDMC), Brigham and Women’s Hospital, and Boston Medical Center, all of whom underwent Ex-CMR at BIDMC between April 2022 and April 2025. All participants provided written informed consent. A prior right heart catheterization (RHC) and iCPET were required, with no intervening cardiac hospitalizations between invasive testing and Ex-CMR, or a major change in cardiac medications relevant to cardiovascular status. The combined RHC/iCPET was performed at each institution using its standard clinical protocol as part of routine patient care. RHC/iCPET procedures were performed per institutional standards; protocol details and measurement methods are summarized in Table S1**.**

Patients were categorized into two predefined clinical groups as follows: (a) cardiac dyspnea, and (b) non-cardiac dyspnea, based on established diagnostic criteria [4], [7], [24], [25], [26], [27], [28], [29]. To minimize referral bias, we applied consistent thresholds for mean pulmonary artery wedge pressure (mPAWP) and the slope of PAWP relative to cardiac output (ΔPAWP/ΔCO). The cardiac dyspnea group included patients meeting any of the following: rest mPAWP ≥15 mmHg, stress mPAWP ≥25 mmHg, ΔPAWP/ΔCO>2, or those with exertional symptoms and CMR imaging findings consistent with stage B HFpEF without recorded definitive extracardiac causes of dyspnea [29], [30]. HFpEF stage B was defined in at-risk patients with preserved LVEF and either reduced LV global longitudinal strain or concentric hypertrophy (excluding HCM criteria) on rest CMR, despite normal mPAWP on RHC or iCPET, consistent with an early phase of cardiac dysfunction [4], [7]. CMR cutoffs used in this study follow contemporary published criteria [7], [26], [28]. Cardiac dyspnea patients could have post-capillary pulmonary artery hypertension secondary to left heart disease [31]. Non-cardiac dyspnea group was defined by invasively confirmed normal hemodynamic profiles (normal rest and stress mPAWP and ΔPAWP/ΔCO≤2), no prior cardiac disease, normal LVEF, normal LV global longitudinal strain, and no concentric hypertrophy.

Exclusion criteria included moderate or greater valvulopathy, LVEF ≤40%, hypertrophic cardiomyopathy, cardiac amyloidosis, cardiac sarcoidosis, coronary artery disease (CAD) requiring intervention, and cor pulmonale. Patients with incomplete cine captures, premature ventricular contractions, or non-sinus rhythm during scanning were excluded from analysis.

A cohort of healthy subjects was also recruited to undergo two Ex-CMR visits to assess the MDI reproducibility. Healthy subjects were individuals leading an active lifestyle with no cardiac conditions and normal rest CMR.

### 2.4 Exercise protocol

**Fig. 2:**
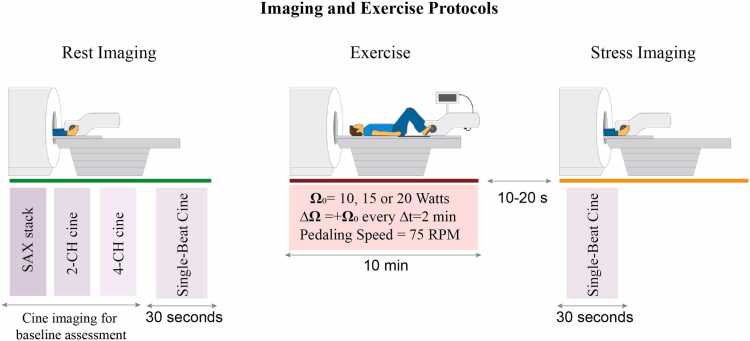
Imaging and exercise protocols. Exercise protocol: initial workload (Ω0) set at 10, 15, or 20 Watts, with workload increments (∆Ω) equal to the initial workload (Ω0) every 2 min (∆t). Pedaling speed was maintained at 75 RPM. *Ex-CMR* exercise cardiovascular magnetic resonance, *SAX* short axis view, *2-CH* 2-chamber view, *4-CH* 4-chamber view, *RPM* revolutions per minute

The exercise protocol has been previously described [17] and is shown in Fig. 2. A CMR-compatible supine ergometer (Lode, Groningen, the Netherlands) attached to the scanner table was used. Exercise was performed outside the magnet bore with the patient positioned on the scanner table, allowing adequate knee movement for cycling across all participants, ensuring protocol consistency, and facilitating effective patient monitoring and communication. The work rate began at 10–20 W and increased in fixed increments, equal to the initial workload, every 2 min at 75 revolutions per minute. The initial workload was determined based on clinical judgment by a study nurse and confirmed by the supervising cardiologist. This decision considered the patient’s performance in a 6-minute walk test conducted immediately before Ex-CMR, as well as their functional capacity, reported in METs from the iCPET. After 10 min or upon exhaustion, whichever came first, subjects were immediately repositioned for stress imaging. Exercise intensity was rated on a 6-point scale from “easy” to “very hard.” Heart rate (HR) and blood pressure were monitored.

### 2.5 Image acquisition and analysis

Fig. 2 shows the imaging protocol. Imaging was conducted on a 3T CMR system (MAGNETOM Vida, Siemens Healthineers, Erlangen, Germany). Cine images were acquired using a balanced steady-state free precession readout. The imaging techniques, parameters, accuracy, and reproducibility were previously described [17], [18]. Baseline CMR characteristics were assessed using a multi-breath-hold, k-space-segmented cine [18]. For Ex-CMR, two cine imaging datasets were acquired for each subject—one at rest (prior to exercise) and the other immediately post-exercise (10–20 s after cessation of exercise). These are referred to as rest and stress imaging, respectively. The Ex-CMR cine imaging used a free-breathing, prospectively ECG-triggered, highly accelerated, multi-slice single-beat cine acquisition (referred to as single-beat cine) with a spatiotemporal resolution of 1.9 × 1.9 mm² and 37 ms [17], [18]. High acceleration was achieved by using compressed sensing image reconstruction followed by applying resolution enhancement generative adversarial inline neural network (REGAIN) with a 14.8-fold acceleration compared to fully sampled k-space. T1 mapping used a modified Look-Locker inversion recovery (MOLLI) breath-hold sequence, acquiring three short-axis slices (base, mid, apex) with voxel size 1.8 × 1.8 × 8 mm³, repetition time 2.5 ms, echo time 1.0 ms, and generalized autocalibrating partial parallel acquisition (GRAPPA) acceleration rate of 2. A cardiologist (F.G) with eight years of experience in CMR analysis measured rest and stress LVEDV, LVESV, LVSV, and LV mass using CVIi42 (version 6.0.2, Circle Cardiovascular Imaging Inc., Calgary, Alberta, Canada), blinded to the patient cohort. These CMR measurements, along with workload from exercise, were used to calculate MDI. Left atrium (LA) volumes were quantified using the biplane area–length method, and peak longitudinal strains of the LV and LA were assessed using feature tracking. CO were calculated as SV × HR. MDI scan–re-scan reproducibility was assessed in a cohort of healthy subjects over a one-year interval. Inter-observer reproducibility was assessed by a reader with 5 years of experience in CMR (M.A.M) on 10% of the data, and intra-observer reproducibility was evaluated on the same sample.

### 2.6 Statistical analysis

Continuous variables were presented as mean ± standard deviation and compared using the Mann-Whitney nonparametric test, as appropriate. Categorical variables were expressed as counts and percentages. Paired non-normally distributed variables were compared using the Wilcoxon matched-pairs signed-rank test. Univariable logistic regression was used to evaluate the association between demographic, clinical, and resting CMR variables and the presence of cardiac vs. non-cardiac dyspnea. Variables with statistically significant odds ratios (ORs) were considered candidates for inclusion in a multivariable base model, constructed in accordance with the event-per-variable rule. This base model incorporated conventional clinical and CMR parameters. To assess the incremental diagnostic value of the MDI, we developed a second model by adding MDI to the base model and evaluated changes in ORs and model performance. The diagnostic performance of three models was compared using receiver operating characteristic (ROC) analysis as follows: (1) the base model, (2) MDI alone, and (3) the base model plus MDI. Area under the curve (AUC) values were computed, and pairwise differences were assessed using the DeLong test. To evaluate whether MDI demonstrated superior discriminative performance compared to its components or their simple interactions, ROC analyses were repeated for the following variables: (1) mean SVi (average of rest and stress SVi), (2) workload (W), (3) LV mass, (4) mean SVi / LV mass, and (5) mean SVi × W. AUC values were calculated and compared to that of MDI using the DeLong test. The optimal cut-off for MDI was identified using the Youden index. The correlation between MDI and the VO₂ max index was also assessed using Pearson’s correlation coefficient. Correlation coefficients were clinically interpreted as follows: correlations <0.20 as very weak, correlations between 0.20–0.39 as weak, correlations between 0.40–0.59 as moderate, correlations between 0.60–0.79 as moderate-strong, and correlations >0.80 as strong [32]. Reproducibility and measurement agreements were assessed using intraclass correlation coefficient (ICC), Bland-Altman analysis, and linear regression, as appropriate. All tests of significance were two-sided and assessed at α <0.05. Analyses were performed using MedCalc (version 23.0.9, MedCalc Software, Ostend, Belgium), Prism (version 10.1.0; GraphPad Software, San Diego, California, USA), and Python (version 3.12) under the supervision of a senior biostatistician (L.N.).

## 3 Results

### 3.1 Patients' clinical and rest CMR characteristics

**Fig. 3:**
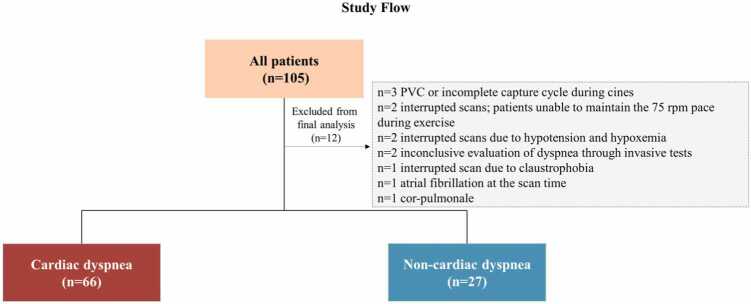
Flow of study cohorts and excluded subjects. *PVC* premature ventricular contraction

93 patients (58 ± 12 years, 33% (31/93) male) were included in the final analyses (Fig. 3). 66 patients (61 ± 11 years, 33% (22/66)male) met the criteria for cardiac dyspnea, while 27 patients (51 ± 11 years, 33% (9/27) male) met the criteria for non-cardiac dyspnea. The median time between invasive testing and Ex-CMR was 2 months, with no report of acute cardiac hospitalization or instability between invasive testing and Ex-CMR. Exactly 15 healthy subjects (48 ± 14 years, 40% male) underwent 2 Ex-CMR studies to assess reproducibility. Table 1 summarizes patients’ clinical and invasive hemodynamic characteristics. Cardiovascular risk factors were more prevalent among patients with cardiac dyspnea as follows: 68% (45/66) had arterial hypertension, 68% (45/66) dyslipidemia, and 21% (14/66) diabetes mellitus. Among patients with cardiac dyspnea, 27% (18/66), 33% (22/66), and 48% (32/66) had rest mPAWP≥15 mmHg, stress mPAWP≥25 mmHg, and ΔPAWP/ΔCO>2 mmHg/L/min, respectively. VO₂ max index data are presented as both continuous and categorical variables (Table 1), the latter classified according to the Weber class [33]. The VO₂ max index was 15.3 ± 4.9 mL/kg/min in patients with cardiac dyspnea and 23.5 ± 6.9 mL/kg/min in those with non-cardiac dyspnea (p<0.0001). Table 2 summarizes the rest CMR characteristics of both groups. No significant differences were observed in traditional resting CMR markers, including LV volumes, LVEF, and CO, between groups. Among cardiac patients, none exhibited an increased LV mass index or systolic anterior motion of the mitral valve with LV outflow tract gradient; however, mild septal LV hypertrophy was observed in 42% (28/66).

**Table 1:** Patient characteristics.

	Cardiac dyspnea (n = 66)	Non-cardiac dyspnea (n = 27)	p value
Age (y)	61±11	51±11	<0.0001
Male n (%)	22 (33%)	9 (33%)	>0.9999
Height (cm)	167±10	172±7	0.004
Weight (kg)	85±18	79±14	0.150
BMI (kg/m^2^)	31±6	27±4	0.001
BSA (m^2^)	2.0±0.3	1.9±0.2	0.609
Heart rate (bpm)	68±14	63±9	0.152
Systolic BP (mmHg)	125±19	120±14	0.039
Diastolic BP (mmHg)	73±11	72±8	0.697
Hypertension	45 (68%)	7 (26%)	0.0004
Dyslipidemia	45 (68%)	5 (19%)	<0.0001
Diabetes	14 (21%)	1 (4%)	0.059
COPD	3 (4%)	2 (7%)	0.626
Asthma	12 (18%)	7 (26%)	0.408
Smoking	17(26%)	7 (26%)	>0.9999
NYHA Class			
I	0	4 (15%)	0.006
II	29 (44%)	18 (67%)	0.067
III	31 (47%)	5 (18%)	0.011
III-IV	6 (9%)	0	0.176
RHC and iCPET Data			
Rest mPAWP ≥15 (mmHg)*	18 (27%)	0	n/a§
Stress mPAWP≥25 (mmHg)†	22 (33%)	0	n/a§
ΔPAWP/ΔCO>2 (mmHg/L/min)‡	32 (48%)	0	n/a§
VO_2_ max index (mL/kg/min)	15.3±4.9	23.5±6.9	<0.0001
Weber class A (>20 mL/kg/min)	12 (18%)	18 (67%)	<0.0001
Weber class B (16–20 mL/kg/min)	11 (17%)	4 (15%)	>0.9999
Weber class C(10–15.9 mL/kg/min)	33 (50%)	5 (18%)	0.006
Weber class D <10 mL/kg/min	10 (15%)	0	0.032
Medications			
β blocker	20 (30%)	3 (11%)	0.065
SGLT2i	11 (17%)	0	0.030
ACEi or ARB	27 (41%)	2 (7%)	0.001
ARNI	1 (2%)	0	>0.9999
MRAs	4 (6%)	0	0.319
Diuretics	21 (32%)	0	0.0003

Continuous data are shown as mean ± SD; categorical data as number (%). ^* † ‡^There is an overlap between patients meeting any of the criteria for cardiac dyspnea; however, one criterion was sufficient for classification. *ACEi* angiotensin-converting enzyme inhibitor, *ARB* angiotensin II receptor blocker, *ARNI* angiotensin receptor neprilysin inhibitor, *BMI* body mass index, *BP* blood pressure, *BSA* body surface area, *CO* cardiac output, *COPD* chronic obstructive pulmonary disease, *iCPET* invasive cardiopulmonary exercise testing, *MRAs* mineralocorticoid receptor antagonists, *mPAWP* mean pulmonary artery wedge pressure, *NYHA* New York Heart Association, *RHC* right heart catheterization, *SGLT2i* sodium-glucose co-transporter 2 inhibitor

* measured during RHC

§ These markers were used to define cohorts; p-value not reported

† measured during iCPET at peak exercise

‡ ratio of the difference in PAWP to the difference in CO, derived from iCPET

**Table 2:** CMR characteristics.

	Cardiac dyspnea (n = 66)	Non-cardiac dyspnea (n = 27)	p value
LVEDV (mL)	139±34	148±36	0.347
LVEDVi (mL/m^2^)	70±14	77±15	0.154
LVESV (mL)	55±18	59±16	0.295
LVESVi (mL/m^2^)	28±8	30±7	0.143
LVSV (mL)	84±24	89±22	0.133
LVSVi (mL/m^2^)	43±10	46±9	0.112
LVCO (L/min)	5.6±1.4	5.5±0.9	0.780
CI (L/min/m^2^)	2.8±0.5	2.8±0.4	0.700
LVEF (%)	61±8	60±4	0.414
LV mass (g)	94±27	85±21	0.146
LV mass index (g/m^2^)	47±10	44±8	0.124
Native T1 (ms)	1257±62	1254±40	0.502
LAV (mL)	71±24	63±22	0.068
LAVi (mL/m^2^)	36±12	32±11	0.092
LA peak strain (%)	39±14	52±10	<0.0001
Structural Heart Disease			
Ventricular Systolic Function			
LVGLS≥ −15%*	37 (59%)	0	-
Morphology			
Septal LVH†	28 (42%)	0	-
Hypertrophy‡	0	0	-

Continuous data are shown as mean ± SD; categorical data as number (%).

All indexed values (denoted by 'i') are normalized to body surface area.

Structural heart disease followed guideline-defined morphological or functional anomalies using established CMR cutoffs, and as per the study design was present only in the cardiac dyspnea. *CI* cardiac output index, *CMR* cardiovascular magnetic resonance, *CO* cardiac output, *EDV* end-diastolic volume, *EF* ejection fraction, *ESV* end-systolic volume, *GLS* global longitudinal strain, *LA* left atrium, *LAV* left atrium volume, *LV* left ventricle, *SV* stroke volume

* Strains were measured using feature tracking

† Wall thickness: male ≥1.2 cm, female ≥1 cm

‡ LV mass index: male ≥83 g/m^2^, female ≥65 g/m^2^

### 3.2 Exercise performance

Table 3 summarizes patients' exercise performance during Ex-CMR. HR increased by 38 ± 17 bpm and 51 ± 16 bpm, and systolic blood pressure rose by 35 ± 18 mmHg and 38 ± 16 mmHg in the cardiac and non-cardiac dyspnea groups, respectively. Patients with cardiac dyspnea exercised for 8.2 ± 2.1 min, reaching a maximum workload of 49 ± 17 W, while those with non-cardiac dyspnea exercised for 9.2 ± 1.3 min, achieving 69 ± 15 W. Patients unable to complete the first stage (<10 W) were excluded. Around 62% (58/93) exercised for ≥9 min, 52% (48/93) completed the full 10-minute protocol, ∼10% (9/93) stopped for safety reasons (e.g., excessive blood pressure or oxygen desaturation), and the remaining participants stopped early due to fatigue or dyspnea. The non-cardiac dyspnea group performed better in W and ΔHR; however, there were no significant differences in the percentage of age-predicted maximum heart rate (%APMHR) (p = 0.518), maximum rate-pressure product (RPP) (p = 0.509), or exercise duration (p = 0.055). Patients’ subjective assessments of exercise intensity did not differ significantly between cohorts (p = 0.342) (Fig. S1)**.** The cardiovascular state remained stressed during stress imaging compared to rest, as evidenced by elevated HR and RPP across all participants **(**Figs. S2 and S3**)**. Fig. S4 shows the breakdown of maximum achieved watts and the correlation between MDI and workload. Exercise performance and hemodynamic responses of the 15 healthy subjects used to assess MDI reproducibility are reported in Table S2. The MDI in healthy subjects was 49.9 ± 11.9 mL·W/g/m².

**Table 3:** Exercise performance, MDI, and its components.

	Cardiac dyspnea (n = 66)	Non-cardiac dyspnea (n = 27)	p value
Response to Exercise			
Duration (min)	8.2±2.1	9.2±1.3	0.055
Max. achieved workload (W)	49±17	69±15	<0.0001
ΔHR (bpm)	38±17	51±16	0.0006
ΔHR (%)	57±30	80±29	0.0005
(%) of age-predicted max HR	69±11	70±11	0.518
Δ Systolic BP (mmHg)	35±18	38±16	0.396
Δ Diastolic BP (mmHg)	18±13	16±11	0.571
Stress RPP	17244±3919	17838± 3216	0.509
Stress HR	109±19	118±17	0.023
MDI and Its Components*			
MDI (mL·W/g/m²)	25.9±9.5	45.1±10.7	<0.0001
LV mass (g)	94±27	85±21	0.146
Mean of rest and stress SVi (mL/m²)	48±9	54±12	0.007
Mean of rest and stress SVi/LV mass (mL/g/m²)	0.54±0.1	0.66±0.1	0.0002
Mean of rest and stress SVi × Workload (mL·W/m²)	2400±1070	3827±1353	<0.0001

Continuous data are shown as mean ± SD; categorical data as number (%).

*BP* blood pressure, *HR* heart rate, *LV* left ventricle, *MDI* myocardial dynamic index, *RPP* rate pressure product calculated as HR × systolic BP, *SVi* stroke volume index. Absolute ΔHR is based on HR change from rest to maximum exercise. ΔHR (%) is the percentage change.

* Workload, as one of the MDI components, is mentioned in earlier rows of this table and not repeated to avoid redundancy

## 4 MDI

**Fig. 4:**
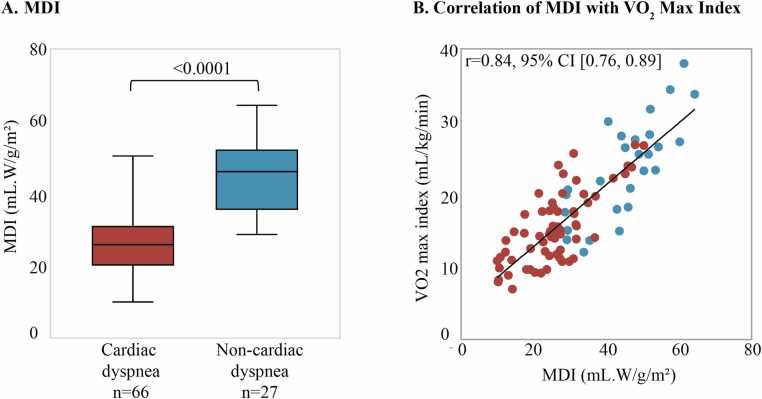
A. MDI across cohorts. MDI was significantly reduced in patients with cardiac dyspnea. B. Pearson correlation between the MDI and VO₂ max index, demonstrating a linear relationship. *MDI* myocardial dynamic index

MDI was 25.9±9.5 mL·W/g/m² in the cardiac dyspnea group and 45.1 ± 10.7 mL·W/g/m² in the non-cardiac dyspnea group (p<0.0001) (Fig. 4**A**). A strong correlation between MDI and VO₂ max index was observed (r = 0.84, [95% CI: 0.76, 0.89], p<0.0001) (Fig. 4**B**). Given the variation due to iCPET mode (upright, n = 73; supine, n = 20), subset analyses were performed to assess the correlation between these two markers. These analyses demonstrated consistent results across modes: Upright: r = 0.81, [95% CI: 0.72, 0.88], p<0.0001; Supine: r = 0.77, [95% CI: 0.50, 0.90], p<0.0001. In addition, we conducted an exploratory sensitivity check by retrospectively evaluating the VO₂ max index threshold in our cohort. The cut-off was 19.8 mL/kg/min, with a sensitivity of 82% and specificity of 75%. Table 3 summarizes the MDI and its component values in both groups.

### 4.1 Univariable and multivariable analysis and model performance

**Fig. 5:**
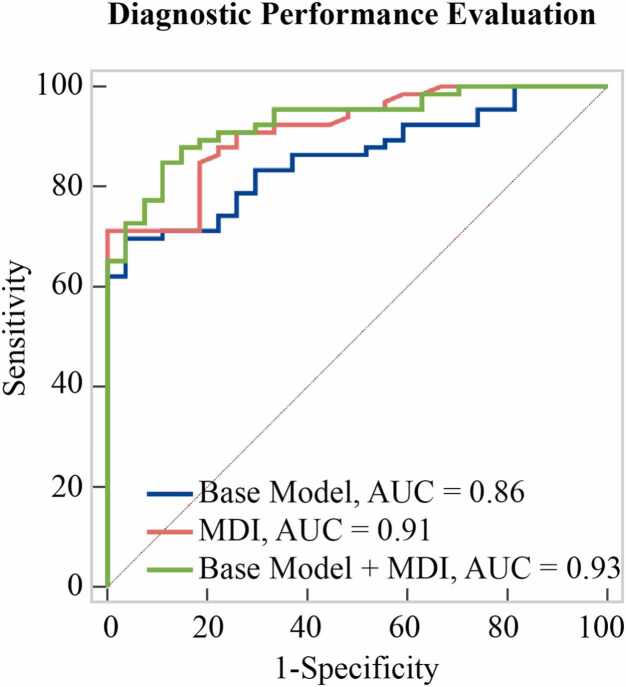
ROC curves evaluating the diagnostic performance of the base model, MDI alone, and the base model combined with MDI for distinguishing cardiac from non-cardiac dyspnea. The base model included age, BMI, NYHA class, and LA peak strain. The addition of MDI to the base model significantly improved discriminative performance (AUC increased from 0.86 to 0.93, p = 0.012, DeLong test). *ROC* receiver operating characteristic, *BMI* body mass index, *NYHA* New York Heart Association, *LA* left atrial, *AUC* area under the curve

**Fig. 6:**
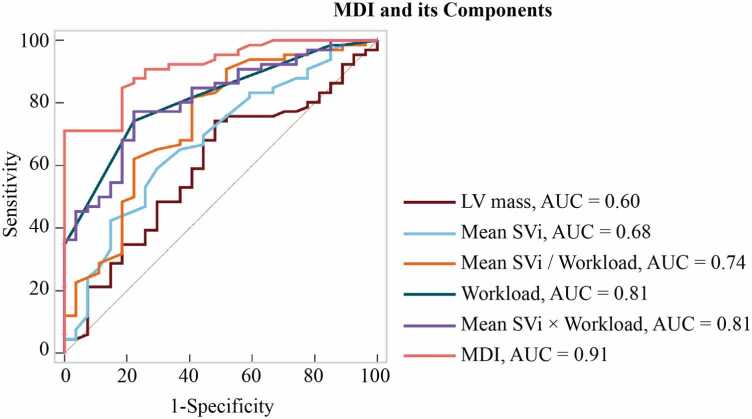
Diagnostic performance of MDI compared to its individual components and interaction-based alternatives. Mean SVi refers to the average of rest and stress stroke volume index. ROC curves illustrate the ability of MDI, its individual components (LV mass, mean SVi, workload), and simple interaction terms (mean SVi/workload and mean SVi × workload) to differentiate cardiac from non-cardiac dyspnea. The AUC was highest for MDI (AUC = 0.91), indicating superior discriminative performance. *MDI* myocardial dynamic index, *SVi* stroke volume indexed to body surface area, *LV* left ventricle, *ROC* receiver operating characteristic, *AUC* area under the curve

In univariable analysis, older age (OR: 1.08 [95% CI: 1.03, 1.13]), higher BMI (OR: 1.15 [95% CI: 1.04, 1.27]), higher NYHA class (OR: 5.80 [95% CI: 2.18, 15.42]), and lower LA peak strain (OR: 0.92 [95% CI: 0.88, 0.97]) were associated with cardiac dyspnea (Table 4). These variables were included in the base multivariable model. Among these, NYHA class (OR: 3.81 [95% CI: 1.25, 11.63]) and LA peak strain (OR: 0.94 [95% CI: 0.89, 0.98]) remained independently associated with cardiac dyspnea (Table 5). MDI alone was associated with cardiac dyspnea (OR: 0.85 [95% CI: 0.79, 0.90]), and when added to the base model, it emerged as the only independent predictor (OR: 0.86 [95% CI: 0.79, 0.93]) (Table 5). Diagnostic performance improved significantly with the inclusion of MDI, increasing the AUC from 0.86 [95% CI: 0.77, 0.92] (base model) to 0.93 [95% CI: 0.86, 0.97] (base model + MDI; p = 0.012 by DeLong test) (Table 6, Fig. 5). A model using MDI alone yielded an AUC of 0.91 [95% CI: 0.84, 0.96], suggesting that MDI alone captures much of the discriminative information provided by the base model (Table 6). For MDI, Youden index analysis identified a cut-off of ≤28.2 mL·W/g/m² (sensitivity 71%, specificity 100%). However, given that 100% specificity does not reflect the clinical variability in our population, the clinically relevant cut-off was set at ≤31.9 mL·W/g/m², balancing false negatives and false positives, with a sensitivity of 85% and specificity of 81% (Table S3). The AUC for MDI was higher than that of each of its components and their interaction (Fig. 6, Table S4).

**Table 4:** Univariable logistic regression: assessing the association with cardiac dyspnea.

Variable	OR	[95%]	p value
Age (y)	1.08	[1.03, 1.13]	0.0006
Sex: Male (vs. Female)	1.00	[0.39, 2.59]	1.000
Systolic BP (mmHg)	1.02	[0.99, 1.04]	0.216
HR (bpm)	1.04	[1.00, 1.08]	0.069
BMI (kg/m^2^)	1.15	[1.04, 1.27]	0.006
NYHA Class	5.80	[2.18, 15.42]	0.0004
LV EDV (mL)	0.99	[0.98, 1.01]	0.266
LV EDVi (mL/m^2^)	0.97	[0.94, 1.00]	0.071
LV ESV (mL)	0.99	[0.96, 1.01]	0.342
LV ESVi (mL/m^2^)	0.96	[0.91, 1.02]	0.167
LV SV (mL)	0.99	[0.97, 1.01]	0.335
LV SVi (mL/m^2^)	0.96	[0.92, 1.01]	0.107
LV EF (%)	1.01	[0.94, 1.08]	0.842
CO (L/min)	1.08	[0.76, 1.53]	0.681
CI (L/min/m^2^)	0.95	[0.38, 2.36]	0.909
LV mass (g)	1.02	[1.00, 1.04]	0.132
LV mass index (g/m^2^)	1.04	[0.99, 1.10]	0.132
LAV (mL)	1.02	[1.00, 1.04]	0.115
LAVi	1.03	[0.99, 1.08]	0.113
LA peak strain (%)	0.92	[0.88, 0.97]	0.0005
T1 native (ms)	1.00	[0.99, 1.01]	0.795

OR and 95% confidence interval (CI) for variables associated with cardiac dyspnea. All indexed values (denoted by “i”) are normalized to body surface area.

*BP* blood pressure, *BMI* body mass index, *CI* cardiac output index, *CO* cardiac output, *EDV* end-diastolic volume, *EF* ejection fraction, *ESV* end-systolic volume, *HR* heart rate, *LA* left atrium, *LAV* left atrium volume, *LV* left ventricle, *NYHA* New York Heart Association, *OR* odds ratio, *SV* stroke volume. LV longitudinal strain and septal LVH, used in cohort definition, were not included in model construction to avoid incorporation bias and overestimation of associations.

**Table 5:** Univariable and multivariable logistic regression analyses.

Variable	Univariable analysis OR [95% CI]	Multivariable analysis	
		Base Model OR [95% CI]	Base Model + MDI OR [95% CI]
Age (y)	1.08 [1.03, 1.13]	1.05 [1.00, 1.11]	1.04 [0.98, 1.11]
BMI (kg/m^2^)	1.15 [1.04, 1.27]	1.10 [0.98, 1.22]	1.03 [0.91, 1.16]
NYHA Class	5.80 [2.18, 15.42]	3.81 [1.25, 11.63]	1.00 [0.20, 4.93]
LA peak strain (%)	0.92 [0.88, 0.97]	0.94 [0.89, 0.98]	0.95 [0.90, 1.00]
MDI (mL·W/g/m²)	0.85 [0.79, 0.90]	-	0.86 [0.79, 0.93]

OR and 95% CI are reported for each variable included in the base model and in the base model with MDI. *BMI* body mass index, *LA* left atrium, *MDI* myocardial dynamic index, *NYHA* New York Heart Association, *OR* odds ratio, *CI* confidence interval

**Table 6:** Diagnostic performance of models.

Models	AUC [95% CI]	p-value (DeLong Test; vs. base model)
Base Model (Age, BMI, NYHA, LA peak strain)	0.86 [0.77, 0.92]	-
MDI alone	0.91 [0.84, 0.96]	0.126
Base Model + MDI	0.93 [0.86, 0.97]	0.012

AUC with 95% confidence intervals (CI) and pairwise comparisons using the DeLong test are shown for the base model, MDI alone, and the base model with MDI. *AUC* area under the ROC curve, *NYHA* New York Association, *BMI* body mass index, *LA* left atrium, *MDI* myocardial dynamic index

### 4.2 Reproducibility assessments

**Fig. 7:**
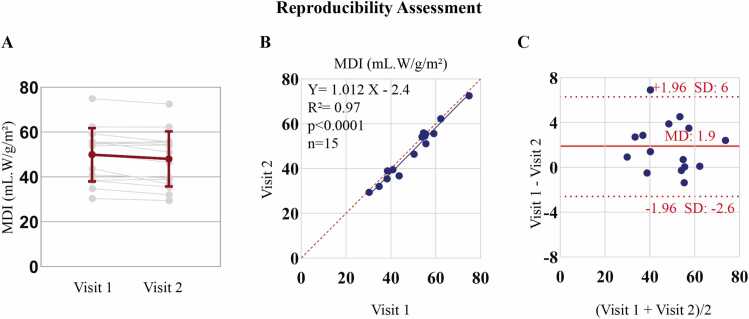
Reproducibility assessment of MDI over one year in 15 healthy subjects (mean time between scans: 11.9 months [Range 9.1–16.6 months]). (A) Line plot showing individual changes from Visit 1 to Visit 2, with mean and standard deviation highlighted. (B) Regression plot illustrating the correlation between Visit 1 and Visit 2 measurements. (C) Bland-Altman plot assessing agreement between the two visits, showing the mean bias and 95% limits of agreement. *MDI* myocardial dynamic index

MDI scan/re-scan reproducibility between two visits was excellent (ICC = 0.96, [95% CI: 0.90, 0.99]. Fig. 7 **A-C** shows individual changes, regression, and Bland-Altman plot, with a mean difference of 1.9 mL·W/g/m² and 95% limits of agreement (−2 to 6 mL·W/g/m²). The ICC for inter- and intra-observer reproducibility of MDI showed good to excellent results: 0.96 [95% CI: 0.85, 0.99] and 0.99 [95% CI: 0.98, 1.0], respectively.

## 5 Discussion

This study proposed and evaluated Ex-CMR MDI as a non-invasive imaging marker. MDI was reduced in patients with cardiac dyspnea compared to those with non-cardiac dyspnea and showed a strong correlation with the VO₂ max index. In multivariable analysis, MDI remained the only independent predictor of cardiac dyspnea after adjustment for clinical variables and LA peak strain, and significantly improved diagnostic performance over the base model. MDI is reproducible, simple to calculate, and feasible for integration into routine Ex-CMR analysis.

### 5.1 Ex-CMR MDI development and its components

MDI is based on established cardio-hemodynamic principles [20], [21], integrating three core components that interact in a complex yet measurable manner. It obviates the need for invasive LV pressure measurements by capturing systolic and diastolic volumetric adaptations to workload-induced cardiac stress. Although LV pressures are not directly included, insights from adaptation and maladaptation of PV curves enable MDI to infer meaningful volume shifts in response to presumed pressure changes from rest to stress [14], [21], [34], [35]. This maladaptation was evident in our study and translated into significantly lower MDI in cardiac dyspnea patients.

Each MDI component serves a distinct role as follows: 1. SV dynamics, indexed to body size, reflect the heart’s volumetric response to workload and are central to oxygen delivery. This response is modulated by LV compliance, which facilitates diastolic volume accommodation, and by contractility, which reduces LVESV [14], [34], [35], [36]. Exercise typically increases LVEDV via faster diastolic pressure decline [35]. However, in HFpEF, even a moderate workload can dramatically elevate LV filling pressures and disrupt the PV relationship, a key mechanism underlying cardiac exertional dyspnea [14], [34], [35]. Similar relaxation abnormalities have been observed in early-stage HFpEF [37]. While LVESV normally decreases with exercise to augment contractility, this response may be blunted in cardiac patients [14], [35]. 2. Workload itself is a key determinant of myocardial oxygen demand, preload, afterload, and CO [9]. As workload increases, the myocardium must adapt its volume dynamics to maintain efficient energy output and oxygen consumption, balancing both internal and external cardiac work. 3. Myocardial mass influences both systolic and diastolic function and enhances the diagnostic utility of quantitative imaging markers when integrated into equations [23], [38], [39], [40], [41], [42]. A higher LV mass-to-cardiac external work ratio is independently associated with prolonged LV relaxation and systolic dysfunction [38]. Additionally, LV mass contributes to internal mechanical and metabolic loads, particularly in conditions like myocardial stiffness [20], [23]. It is central to cardiac remodeling and structural heart disease [4], [43]. Adjusting MDI for myocardial mass accounts for its role in both external and internal work, much of the latter being lost as heat [20]. Our analyses confirmed that MDI provides added value beyond its individual components or their simple interactions with mean of rest and stress SVi.

Direct comparison of MDI with other cardiac indices that reflect oxygen consumption is limited by methodological differences. For example, the invasive PV loop area quantifies mechanical energy per cardiac cycle as the integral of pressure and volume within the loop [19], [21], [22]. The non-invasive pressure-strain loop (PSL) uses the loop area as a surrogate for regional myocardial work and reflects myocardial metabolism [44], [45]. However, PSL relies on an optimal ultrasound window, is load-dependent, and, similar to the rest CMR-derived non-invasive PV loop [46], overlooks diastolic indices, making it less applicable during exercise and less specific for evaluating cardiac dyspnea [47].

### 5.2 Ex-CMR MDI clinical implications

Our findings indicate that MDI adds value beyond clinical variables and LA strain, a surrogate marker of diastolic function, in differentiating cardiac from non-cardiac dyspnea. Ex-CMR is an emerging advanced diagnostic tool, but to date, only a few studies have explored the predictive value of imaging markers for cardiac dyspnea using this modality [16], [48], [49]. In the HFpEF Stress Trial, Backhaus et al. demonstrated that stress LA strain was reduced in HFpEF patients compared to those with non-cardiac dyspnea and was independently associated with cardiac dyspnea [49]. While LA strain remains a robust surrogate of diastolic dysfunction in HFpEF, MDI offers a novel Ex-CMR perspective through an alternative approach. It captures a blunted SV response to physiological stress in patients with cardiac dyspnea, accounts for energy expended against myocardial resistance and body size, and correlates with VO_2_ max index. Guazzi et al. found that limited CO at peak exercise was the strongest determinant of low VO₂ max in heart failure patients [33]. The MDI design enhances sensitivity to SV changes in response to workload, reducing dependence on high-intensity exertion and emphasizing the heart’s response at a given effort level. Hemodynamic abnormalities have frequently been reported within minutes of exercise in HFpEF patients, even at low-to-moderate intensities [24], [30], [37], with 20 W generally considered the threshold at which these anomalies, if present, typically manifest. Our study employed a submaximal exercise protocol, with a median maximum achieved workload of 50 W and no difference in %APMHR or in subjective exercise intensity between cohorts. We observed some variation in maximum workload and excluded participants who could not complete the first stage (<10 W), but we did not exclude patients based on workload or early termination of exercise before reaching 10 min. Compared to the HFpEF Stress Trial, MDI was evaluated in a larger patient cohort encompassing the HFpEF spectrum, from early to advanced stages. A recent study investigating stage B HFpEF indicates that even among patients in whom overt HFpEF has been excluded, a higher burden of HFpEF risk factors and echocardiographic functional and structural abnormalities is strongly associated with aerobic limitations typically seen in overt HFpEF [50]. This highlights the added value of CMR in identifying stage B HFpEF. Our holistic approach, integrating invasive and non-invasive assessments, reflects real-world, guideline-directed clinical practice. The profile of our cardiac patients aligns with conditions such as overt HFpEF, exercise-induced HFpEF, stage B HFpEF, and HFmrEF, clinically challenging phenotypes to manage [4]. However, whether MDI can further distinguish the underlying pathologies within cardiac dyspnea requires thresholding and external validation, and remains to be explored in larger studies.

In light of previous studies, such as that by Le et al. [51], which demonstrated the value of the stress cardiac index, the MDI inherently captures the core volumetric component of CI in its formulation, but does not account for HR in our patient population. This is because we did not ask our patients to withhold beta-blockers, did not directly account for chronotropic incompetence, and employed a submaximal exercise intensity. While this approach ensures reproducibility for studies investigating dyspneic patients, it limits a fair interpretation of stress CI. Further efforts are warranted to optimize MDI, or other Ex-CMR markers, by accounting for HR, beta-blocker use, and chronotropic incompetence.

### 5.3 MDI and VO_2_ max index

The strong correlation between MDI and VO₂ max index supports the physiological validity of MDI as a measure of cardiac performance under increasing oxygen demand, as both incorporate SV in their calculations. VO₂ max reflects multisystem oxygen transport and consumption, making it a robust yet complex biomarker, whereas MDI offers a more targeted assessment of cardiac limitations, particularly in patients with dyspnea. In our study, reduced SV exchange contributed to lower MDI, paralleling prior findings that limited CO predicts low VO₂ max [33]. However, these two markers are not interchangeable; the correlation was intended as a physiological benchmark [44]. Unlike VO₂ max, which requires achieving the respiratory exchange ratio (RER) threshold, MDI remains effective at submaximal exercise, providing a practical tool to detect cardiac dysfunction before invasive testing, though dyspnea may still have multifactorial origins. A limitation of our study is that VO₂ max values were derived from both supine and upright iCPET. However, the correlation remained strong for both exercise modes, and the VO₂ max index threshold for identifying cardiac dyspnea (19.8 mL/kg/min) closely aligned with the upper limit of Weber class B (20 mL/kg/min) [33]. This suggests that the observed correlation between MDI and VO₂ max is robust and unlikely to be substantially biased by the mix of iCPET protocols. Nevertheless, a direct, head-to-head comparison of supine iCPET with supine Ex-CMR-derived MDI, ideally performed within a shorter interval, is warranted.

## 6 Limitations

The Ex-CMR was performed at a single center using the same CMR scanner. We did not investigate patients with multifactorial causes of dyspnea. Some referral bias from participating sites may be present, and the mix of supine and upright iCPET applied at our referral sites adds some heterogeneity, though this reflects routine practice. However, our approach—which integrated both invasive and non-invasive parameters, applied widely accepted clinical cutoffs for classification, and included a spectrum of HFpEF—was designed to mitigate this limitation. Although patients with cardiac dyspnea achieved lower levels of exertion, they reached similar %APMHR and maximum RPP compared to those with non-cardiac dyspnea. A head-to-head comparison of MDI between healthy and patient populations across different exercise protocols, modes, and intensities should be interpreted cautiously. Further studies are needed for external and prospective validation of MDI, to define the optimal workload range, assess MDI across a broader clinical spectrum and in different Ex-CMR protocols with higher intensity or alternative exercise execution, and to evaluate its prognostic value. Our study design did not allow for accurate calculation of the H₂FPEF score in our patients. However, given the very low prevalence of atrial fibrillation (2%) and the descriptive characteristics of our cohort, we postulate that our population represented an intermediate-risk group. This observation warrants further investigation to evaluate MDI performance across different clinical risk profiles and to identify the optimal patient population most likely to benefit from MDI.

## 7 Conclusion

Ex-CMR MDI is a novel non-invasive imaging marker associated with cardiac dyspnea and strongly correlated with the VO₂ max index. It aids in distinguishing cardiac from non-cardiac dyspnea and may offer added diagnostic value beyond conventional clinical and resting imaging parameters.

## Funding

Reza Nezafat receives grant funding from the National Institutes of Health (NIH) R01 HL158077 (Bethesda, MD, USA).

## Author contributions

**F.G.** developed the work-volume loop model, formulated the myocardial dynamic index, conceptualized the study design, performed data collection, image and statistical analysis, and prepared the manuscript. **J.R.** was involved in patient recruitment and consenting. **M.A.M.** performed inter-observer measurements and contributed to the study design. **L.H.N.** oversaw statistical analyses. **C.W.T.**, **J.M.R.**, **D.M.G.**, **D.M.S.**, and **A.B.W.** contributed to patient recruitment and study design. **W.J.M.** and **R.N.** contributed to the study design, data interpretation, and manuscript revision. All authors critically revised the paper and have read and approved the final manuscript.

## Ethics approval and consent

This HIPAA-compliant study was approved by our institutional review board. All participants provided written informed consent.

## Declaration of competing interests

The authors report no conflict of interest.

## Availability of data and materials

Funder requirements for data sharing are not applicable, and data will not be shared or will be available upon request.

## Footnotes

Appendix A Supplementary data associated with this article can be found in the online version at doi:10.1016/j.jocmr.2025.101981.
