# Supplementary material for: Exercise cardiovascular magnetic resonance myocardial dynamic index: A non-invasive imaging marker associated with cardiac dyspnea
Source: J Cardiovasc Magn Reson. 2025 Oct 27;27(2):101981. doi: 10.1016/j.jocmr.2025.101981 (PMC12766608; doi:10.1016/j.jocmr.2025.101981)
Supplement: Supplementary file 1 — Supplementary material [file mmc1.docx]

**Supplementary materials**

| **Table S1 Right Heart Catheterization (RHC) and Invasive Cardiopulmonary Exercise Testing (iCPET) Protocols, and Cardiac Output and VO₂ Max Index Calculation.** |
| --- |
| RHC and iCPET were conducted across three sites: Beth Israel Deaconess Medical Center (BIDMC, n=13), Brigham and Women’s Hospital (BWH, n=60), and Boston Medical Center (BMC, n=20). Relevant technical protocols and measurements for our study are outlined below, with minor protocol variations explicitly stated  RHC was performed via the right internal jugular or right brachial vein, selected at the operator’s discretion based on clinical judgment. A pulmonary artery catheter (French size adjusted per patient) was advanced through the right-sided cardiac chambers into the pulmonary artery and positioned in the pulmonary capillary wedge location. Repositioning was required if the patient’s posture changed between RHC and iCPET. Radial artery cannulation was performed for arterial blood sampling. Hemodynamic measurements were obtained in the appropriate chambers, with blood gas analysis for oximetry and cardiac output determined using the Fick method or assumed Fick method. All pressure recordings were taken at end-expiration.  $CO=\frac{Oxygen Consumption}{Arterial Oxygen Content-Venous Oxygen COntent}$  All hemodynamic measurements for RHC were acquired in the supine position. Exercise testing was performed using an upright (BWH, BIDMC) or a supine (BMC) bike ergometer. A ramp protocol (10–25 W/min) was implemented, maintaining a cycling rate of 60-70 RPM with progressive workload increments until exhaustion. Key hemodynamic indices included rest and stress pulmonary artery wedge pressure (PAWP) and cardiac output (CO). The ratio of change in PAWP and CO (ΔPAWP/ΔCO) was calculated.  Baseline oxygen consumption at rest (supine) was assumed to be 125 mL/min/m², while dynamic oxygen consumption during exercise was directly measured using a metabolic cart. Arterial oxygen saturation was assessed via both point-of-care oximetry and arterial blood gas (ABG) analysis. Maximal VO₂ was determined at peak exercise and indexed to body weight (VO₂ max). Predicted VO₂ (%) for age and sex was calculated based on Wasserman equations, with FRIEND percentiles occasionally reported. Data interpretation required an achieved respiratory exchange ratio (RER) of 1.05–1.1.  $VO2\max=CO\max\times\left( a-v \right)O2 max$ |

| **Table S2 Exercise Performance of Healthy Subjects at Visit 1 and Visit 2** | |  |
| --- | --- | --- |
|  | Visit 1 (n=15) | Visit 2 (n=15) |
| **Response to Exercise** | | |
| Duration (min) | 9 ± 1.7 | 9 ± 1.2 |
| Max. workload (W) | 76 ± 18 | 74 ± 20 |
| Absolute ΔHR (bpm) | 46 ± 10 | 48 ± 15 |
| Relative ΔHR (%)* | 67 ± 15 | 73 ± 23 |
| (%) of age-predicted max HR | 67 ± 8 | 67 ± 10 |
| Stress HR | 115 ± 14 | 114 ± 17 |
| Δ Systolic BP (mmHg) | 34 ± 14 | 36 ± 8 |
| Note. — Continuous data are shown as mean ± SD; categorical data as number (%). BP = blood pressure; HR = heart rate; MDI = myocardial dynamic index. Absolute ΔHR is based on HR change from rest to maximum exercise. *ΔHR (%) is the percentage change. | | |

| **Table S3** **MDI Cut-off Value Derived from Youden Index in ROC Analysis** | | | | | | |
| --- | --- | --- | --- | --- | --- | --- |
| Criterion | Sensitivity | 95% CI | Specificity | 95% CI | + LR | -LR |
| <10 | 0.00 | 0.0 – 5.4 | 100.00 | 87.2 – 100.0 | — | 1.00 |
| ≤28.2 | 71.21 | 58.7 – 81.7 | 100.00 | 87.2 – 100.0 | — | 0.29 |
| ≤29.5 | 71.21 | 58.7 – 81.7 | 81.48 | 61.9 – 93.7 | 3.85 | 0.35 |
| **≤31.9** | **84.85** | **73.9 – 92.5** | **81.48** | **61.9 – 93.7** | **4.58** | **0.19** |
| ≤33.9 | 86.36 | 75.7 – 93.6 | 77.78 | 57.7 – 91.4 | 3.89 | 0.18 |
| ≤35 | 87.88 | 77.5 – 94.6 | 77.78 | 57.7 – 91.4 | 3.95 | 0.16 |
| ≤35.5 | 87.88 | 77.5 – 94.6 | 74.07 | 53.7 – 88.9 | 3.39 | 0.16 |
| ≤37.1 | 90.91 | 81.3 – 96.6 | 74.07 | 53.7 – 88.9 | 3.51 | 0.12 |
| ≤40.6 | 90.91 | 81.3 – 96.6 | 66.67 | 46.0 – 83.5 | 2.73 | 0.14 |
| ≤42 | 92.42 | 83.2 – 97.5 | 66.67 | 46.0 – 83.5 | 2.77 | 0.11 |
| ≤44.2 | 92.42 | 83.2 – 97.5 | 55.56 | 35.3 – 74.5 | 2.08 | 0.14 |
| ≤45.3 | 93.94 | 85.2 – 98.3 | 51.85 | 31.9 – 71.3 | 1.95 | 0.12 |
| ≤46 | 95.45 | 87.3 – 99.1 | 51.85 | 31.9 – 71.3 | 1.98 | 0.088 |
| ≤46.7 | 95.45 | 87.3 – 99.1 | 44.44 | 25.5 – 64.7 | 1.72 | 0.10 |
| ≤47.1 | 96.97 | 89.5 – 99.6 | 44.44 | 25.5 – 64.7 | 1.75 | 0.068 |
| ≤48 | 98.48 | 91.8 – 100.0 | 40.74 | 22.4 – 61.2 | 1.66 | 0.037 |
| ≤49.1 | 98.48 | 91.8 – 100.0 | 37.04 | 19.4 – 57.6 | 1.56 | 0.041 |
| ≤50.4 | 100.00 | 94.6 – 100.0 | 33.33 | 16.5 – 54.0 | 1.50 | 0.00 |
| ≤64.4 | 100.00 | 94.6 – 100.0 | 0.00 | 0.0 – 12.8 | 1.00 | — |
| Note. — CI= confidence interval; + LR= positive likelihood ratio; -LR= negative likelihood ratio;  MDI= myocardial dynamic index; ROC= receiver operating characteristic. Analysis were performed, using MedCalc (version 23.0.9, MedCalc Software, Ostend, Belgium). | | | | | | |

| **Table S4 Performance Evaluation of MDI compared to Its Individual Components, and Its Components’ Interactions** | | |
| --- | --- | --- |
| Variables | AUC [95% CI] | p-value (vs. MDI, DeLong Test) |
| MDI (mL·W/g/m²) | 0.91 [0.84, 0.96] | - |
| LV mass (g) | 0.60 [0.49, 0.70] | <0.0001 |
| Mean of rest and stress SVi (mL/m²) | 0.68 [0.57, 0.77] | <0.0001 |
| Mean of rest and stress SVi /LV mass (mL/g/m²) | 0.74 [0.68, 0.82] | 0.002 |
| Workload (W) | 0.81 [0.72, 0.89] | 0.001 |
| Mean of rest and stress SVi × Workload (mL·W/m²) | 0.81 [0.71, 0.88] | 0.001 |
| Note. — Data represent the area under the ROC curve (AUC) and the 95% confidence interval (CI) of the receiver operating characteristic analysis. Statistical improvement of MDI over other variables was assessed using DeLong’s test for AUC comparison. LV= left ventricle; MDI = myocardial dynamic index. | | |

**
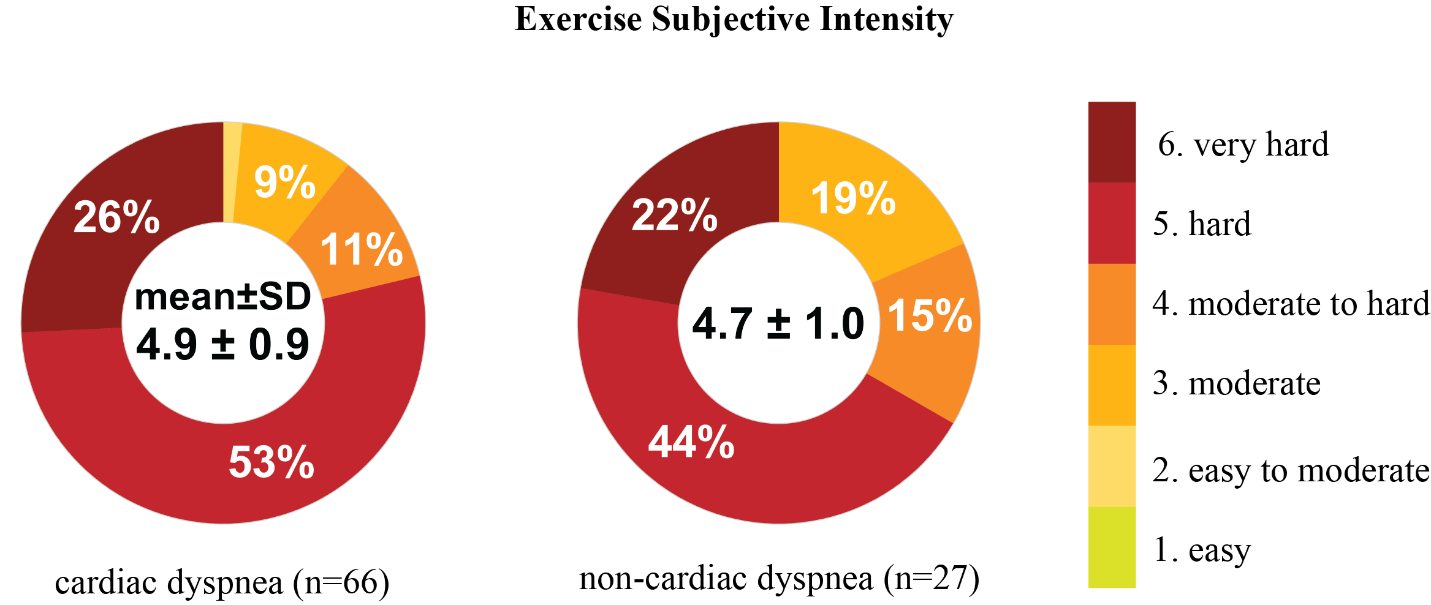
Figure S1.** Exercise subjective intensity. During supervised exercise, at each workload stage, participants were asked to rate their subjective perception of exercise intensity using the following scale: 1. easy, 2. easy to moderate, 3. moderate, 4. moderate to hard, 5. hard, 6. very hard. Note: For values less than 5%, only color-coding is provided.


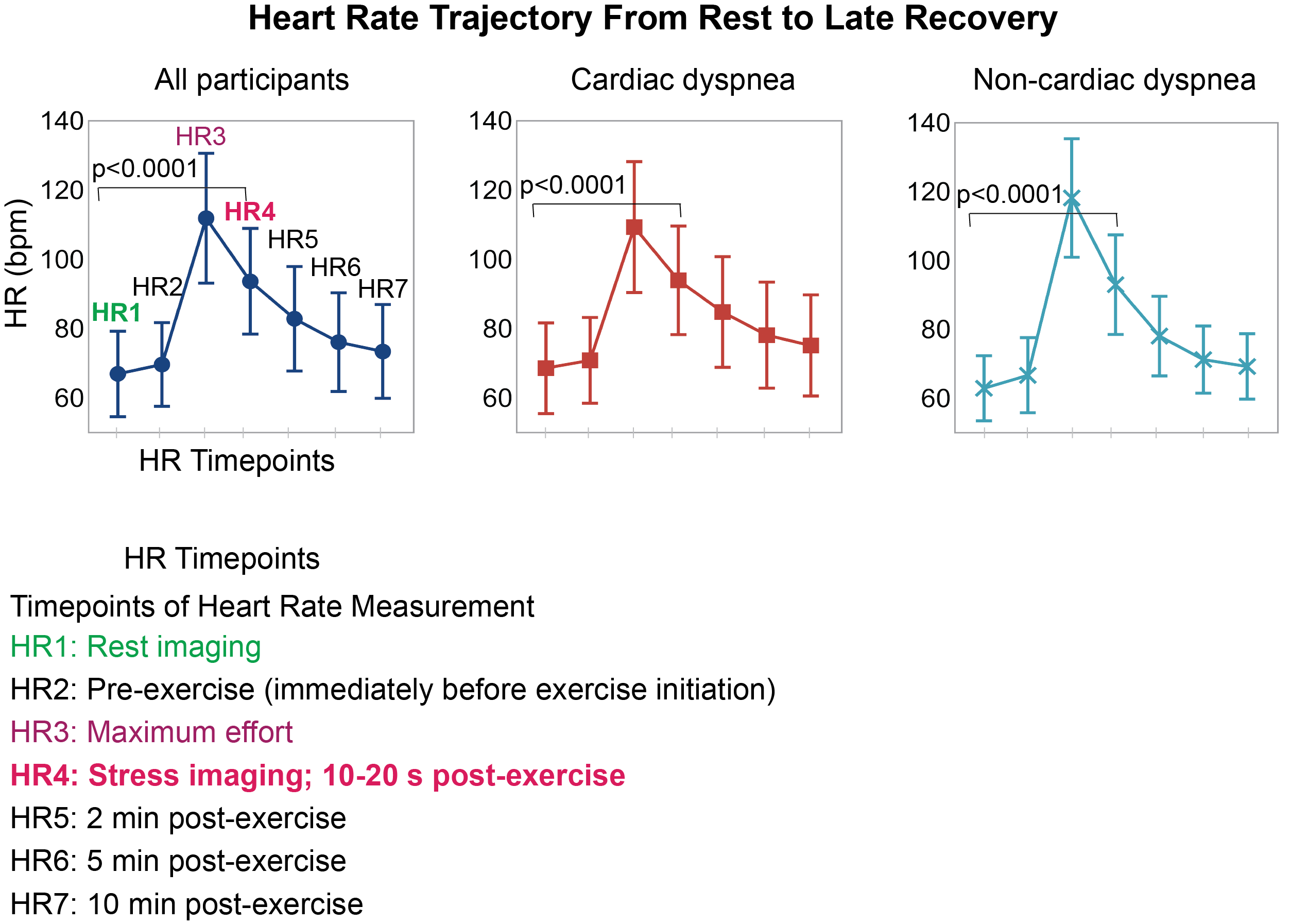


**Figure S2.** Heart rate trajectory from rest to late recovery across study cohorts. HR1 indicates heart rate recorded during rest cine; HR3 indicates peak heart rate during the final exercise stage; HR4 represents heart rate at the time of stress cine acquisition (10–20 seconds post-exercise). HR4 remained significantly elevated compared to HR1.


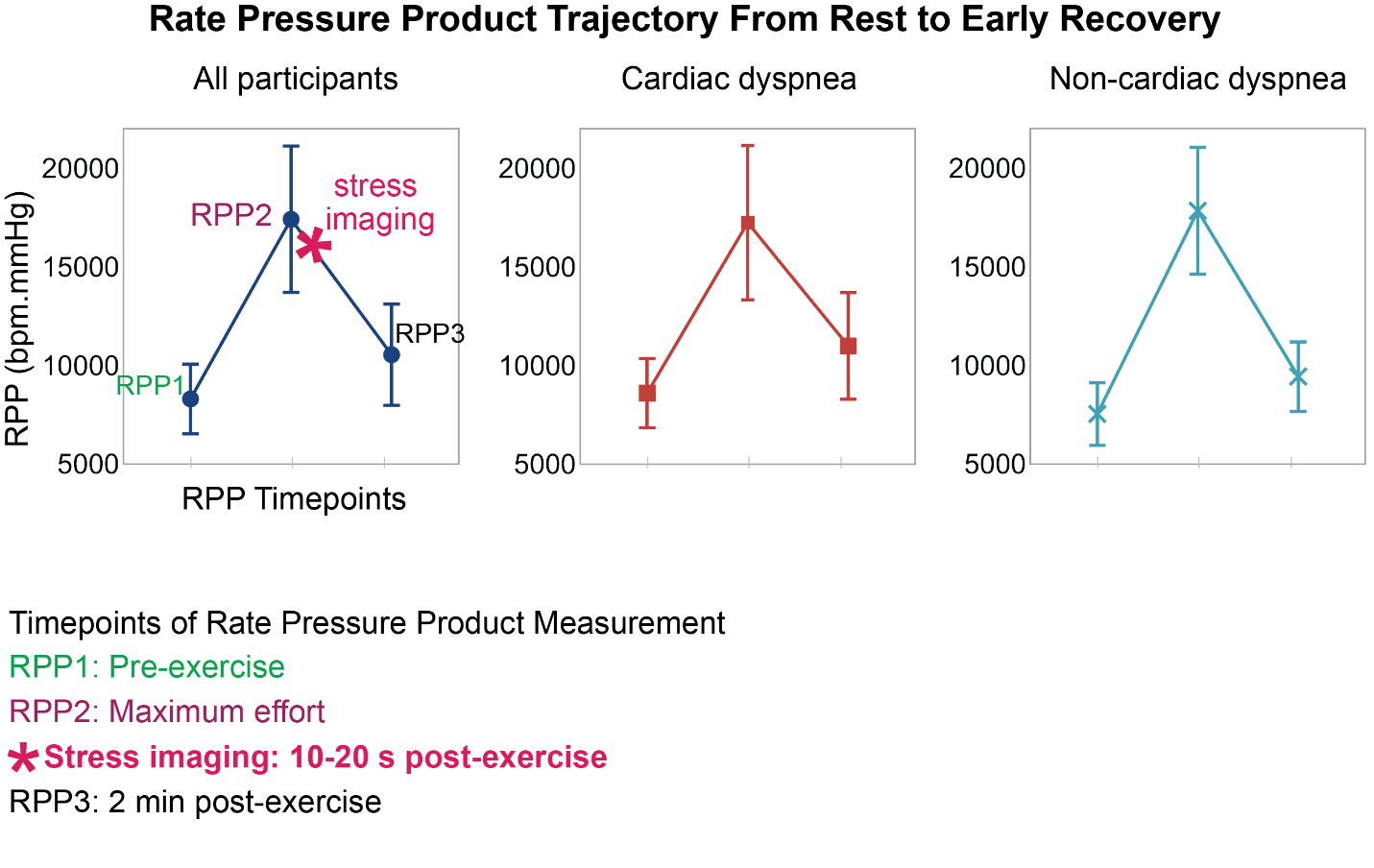


**Figure S3.** Rate pressure product (RPP) trajectory from rest to recovery. The pink bold asterisk marks the time of stress cine acquisition, immediately after cessation of exercise, indicating that the cardiovascular system remained under stress during imaging.


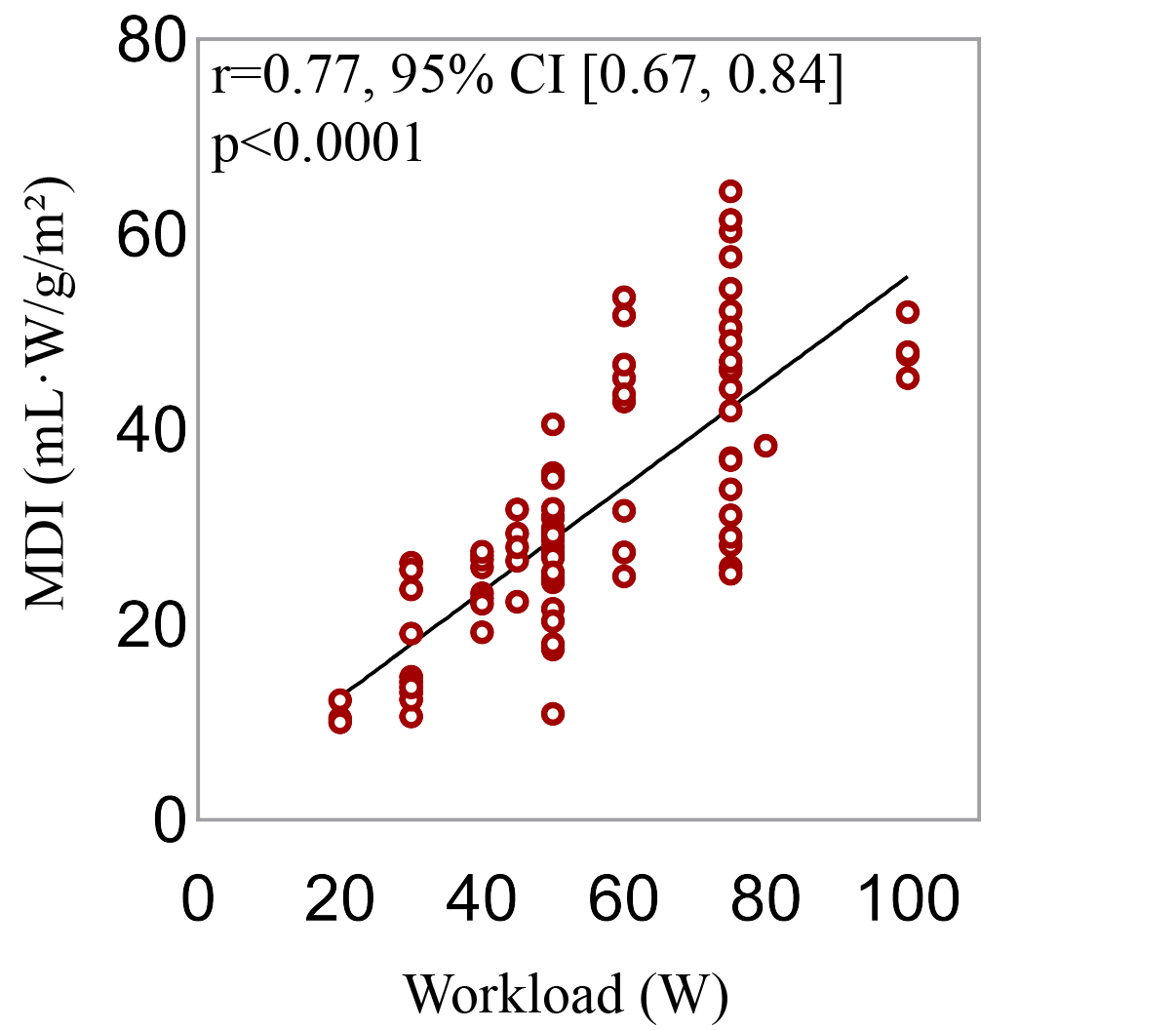


**Figure S4.** Breakdown of maximum achieved workload in all patients and the correlation between MDI and workload.
